# Profound parental bias associated with chromosome 14 acquired uniparental disomy indicates targeting of an imprinted locus

**DOI:** 10.1038/leu.2015.130

**Published:** 2015-07-31

**Authors:** A Chase, W Leung, W Tapper, A V Jones, L Knoops, C Rasi, L A Forsberg, P Guglielmelli, K Zoi, V Hall, L Chiecchio, L Eder-Azanza, C Bryant, L Lannfelt, L Docherty, H E White, J Score, D J G Mackay, A M Vannucchi, J P Dumanski, N C P Cross

**Affiliations:** 1 Wessex Regional Genetics Laboratory, Salisbury NHS Foundation Trust, Salisbury District Hospital, Salisbury, UK; 2 Faculty of Medicine, University of Southampton, Southampton, UK; 3 Hematology unit, Cliniques Universitaires Saint-Luc and de Duve Institute, Université Catholique de Louvain, Brussels, Belgium; 4 Department of Immunology, Genetics and Pathology, Science for Life laboratory, Uppsala University, Uppsala, Sweden; 5 Laboratorio Congiunto MMPC, Department of Experimental and Clinical Medicine, University of Florence, Florence, Italy; 6 Haematology Research Laboratory, Biomedical Research Foundation, Academy of Athens, Athens, Greece; 7 Department of Public Health and Caring Sciences, Uppsala University, Uppsala, Sweden

## Abstract

Acquired uniparental disomy (aUPD) is a common finding in myeloid malignancies and typically acts to convert a somatically acquired heterozygous mutation to homozygosity. We sought to identify the target of chromosome 14 aUPD (aUPD14), a recurrent abnormality in myeloid neoplasms and population cohorts of elderly individuals. We identified 29 cases with aUPD14q that defined a minimal affected region (MAR) of 11.2 Mb running from 14q32.12 to the telomere. Exome sequencing (*n*=7) did not identify recurrently mutated genes, but methylation-specific PCR at the imprinted *MEG3-DLK1* locus located within the MAR demonstrated loss of maternal chromosome 14 and gain of paternal chromosome 14 (*P*<0.0001), with the degree of methylation imbalance correlating with the level of aUPD (*r*=0.76; *P*=0.0001). The absence of driver gene mutations in the exomes of three individuals with aUPD14q but no known haematological disorder suggests that aUPD14q may be sufficient to drive clonal haemopoiesis. Analysis of cases with both aUPD14q and *JAK2* V617F (*n*=11) indicated that aUPD14q may be an early event in some cases but a late event in others. We conclude that aUPD14q is a recurrent abnormality that targets an imprinted locus and may promote clonal haemopoiesis either as an initiating event or as a secondary change.

## Introduction

Uniparental disomy (UPD) refers to the situation in which both copies of a chromosome pair or parts of chromosomes have originated from one parent. Constitutional UPD is associated with developmental disorders caused by the abnormal expression of imprinted genes, that is, genes that are differentially expressed depending on whether they have been maternally or paternally inherited. By contrast, somatically acquired UPD (aUPD) in cancer is a mechanism by which a pre-existing driver mutation (usually somatically acquired) is converted to homozygosity, thereby providing an additional clonal advantage. aUPD may involve whole chromosomes as a result of non-disjunction or, more commonly, whole chromosome arms or terminal segments as a consequence of mitotic recombination. aUPD cannot be detected by conventional cytogenetics but is readily apparent by the finding of somatically acquired long homozygous tracts without change in copy number by genome-wide single nucleotide polymorphism (SNP) analysis.^1^

aUPD is prevalent in myeloid neoplasms: chromosomes 4q, 7q, 9p, 11q and 13q are commonly affected and target mutated *TET2*, *EZH2*, *JAK2*, *CBL* and *FLT3*, respectively.^1^ Several other regions of recurrent aUPD have been identified for which the target is unknown, for example, chromosome 14q aUPD (aUPD14q) is seen in myeloid neoplasms^2^ and is one of the most common abnormalities associated with clonal haemopoiesis in population cohorts of elderly individuals.^3, 4^ Here we show an unexpected and highly significant parental bias associated with aUPD14q, implicating an imprinted locus at 14q32 as the primary target rather than a specific mutated gene.

## Patients and methods

### Study cohorts

Our study comprised two major groups of individuals: (i) patients diagnosed with a myeloproliferative neoplasm (MPN) or myelodysplastic (MDS)/MPN according to standard morphological, haematologic and laboratory criteria; (ii) population cohorts of elderly individuals from Sweden, specifically the Uppsala Longitudinal Study of Adult Men^5^ and the Prospective Investigation of the Vasculature in Uppsala Seniors (PIVUS).^6^ The study was approved by the following ethics committees or review boards: the National Research Ethics Service (UK) Committee South West, the Uppsala Regional Ethical Review Board, the Ethics Committee of the Biomedical Research Foundation of the Academy of Athens, Comitato Etico, Azienda Ospedaliero-Universitaria Careggi, Firenze. Informed consent was obtained according to the Declaration of Helsinki.

### Molecular analysis

Genome wide SNP profiles for MPN and MDS/MPN cases, most of which have been published previously,^2, 7^ were obtained from peripheral blood or bone marrow leucocyte DNA using the Affymetrix SNP 6.0 (Affymetrix, Santa Clara, CA, USA) or Illumina Human OmniExpressExome v1.2 platforms (Illumina, San Diego, CA, USA). Peripheral blood leucocyte profiles for the Swedish population cohorts were obtained using Illumina 2.5M HumanOmni arrays (Illumina) have also been published.^8^ Exome sequencing for MPN and MDS/MPN cases was performed using the Agilent SureSelect kit (Agilent Technologies, Palo Alto, CA, USA) (Human All Exon 50 Mb) and sequenced using an Illumina HiSeq 2000 (Illumina) at the Wellcome Trust Centre for Human Genetics at Oxford, UK. Exome sequencing for the Swedish cases was performed by SciLifeLab, Stockholm, Sweden.

For methylation analysis, DNA was bisulphited (Zymo Research, Ervine, CA, USA) alongside four healthy controls, amplified using forward primers specific for methylated or unmethylated *MEG3* or *NHP2L1* with a common FAM labelled reverse primer and analysed with a 3130xl Genetic Analyser (Applied Biosystems, Foster City, CA, USA) as described.^9^ Loss of heterozygosity analysis at 14q32 was performed using microsatellites D14S553, D14S267, D14S1006, D14S542, D14S292 and D14S1007, again using a 3130xl Genetic Analyser. All primer sequences are listed in Supplementary Table 1.

### SNP array analysis

Array analysis was performed as described.^7, 10, 11^ For each SNP the log R Ratio, a measure of normalised total signal intensity, and B Allele Frequency (BAF), a measure of normalised allelic intensity ratio, were determined using the BeadStudio (Illumina) and pennCNV^12^ software for Illumina and Affymetrix arrays, respectively. Regions of aUPD were identified by BAF segmentation^10^ which excluded non-informative SNPs (SNPs with BAF >0.9 or BAF <0.1 and SNPs where the absolute difference in BAF between preceding and succeeding SNPs is >0.6), mirrored BAF at 0.5 and used circular binary segmentation to identify regions with similar allelic proportions. For heterozygous SNPs, the BAF is the proportion of the total signal (A+B) accounted for by one allele (B). In a mixed population of cells, the segmented mirrored BAF value will be a combination of values of 1 and 0.5 for cells with and without aUPD, respectively. Regions of aUPD were therefore defined as a region of allelic imbalance (segmented mirrored BAF >0.56) with neutral copy number (log R ratio ~0) that extended to the telomere.^12^ For samples with known *JAK2* V617F levels, determined by pyrosequencing,^13^ and BAF for both aUPD9p and aUPD14q aUPD, we were able to calculate both the proportion of cells with aUPD14 and the proportion of cells which were homozygous or heterozygous for *JAK2* V617F. This allowed us to infer the likely order of acquisition of aUPD14 and *JAK2* V617F (see Supplementary Table 2 for detailed calculations).

### Sequence analysis

Analysis of exome sequencing data was as previously described.^14^ On average, the targeted exome of chromosome 14 was sequenced to a depth of 179 ×, and 94.3% of the target bases were covered by at least 20 reads (Supplementary Table 3). For variants passing quality control (read depth ⩾4, alternate read depth ⩾2, phred scaled quality ⩾20, phred scaled base call accuracy ⩾10, strand bias *P*⩾0.0001, base quality bias *P*⩾1e-100, tail bias *P*⩾0.0001 and HWE *P*⩾0.0001), variant allele frequencies were used to approximate BAF and were analysed accordingly to confirm regions of aUPD. The minimally affected region identified in patient E5364 (chr14:94,245,652-qter) was interrogated in all aUPD14q exomes for rare variants that were either novel or had a minor allele frequency of ⩽1% in databases of common variation (1000 genomes, Complete Genomics, Exome Variant Server).

## Results

### Prevalence of aUPD14q

We analysed in house array data on cases with MPN or MDS/MPN and identified aUPD14q extending to the 14q telomere in 25 cases. Of the UK cases (*n*=21), 1/293 (0.3%) had MDS/MPN, 8/563 (1.6%) had *JAK2* V617F negative MPN (7 *CALR* mutated; 1 *MPL* mutated) and 12/1054 (1.1%) had *JAK2* V617F positive MPN, an overall prevalence similar to that identified in other studies of myeloid neoplasms (8/498; 1.6%).^2, 7, 15, 16, 17^ Trisomy chromosome 14 (+14) was seen in 4/293 (1.4%) MDS/MPN cases.^2^ Of the 1641 individuals ⩾70 years of age in the Swedish population-based cohorts, 4 (0.2%) had aUPD14,^8^ similar to the frequency in elderly individuals reported by other larger studies of cases recruited for a variety of genome-wide association studies.^3, 4^ Cases with aUPD14q or other chromosome 14 abnormalities in our study are summarised in Table 1.

### Minimally affected region

**Figure 1:**
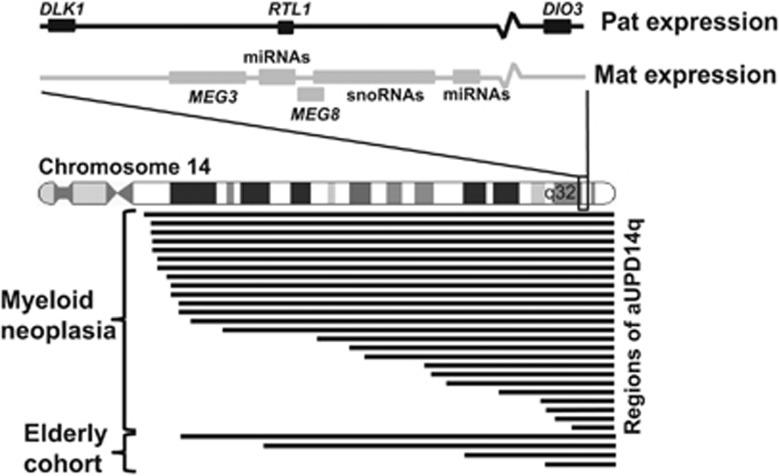
Extent of aUPD14q in the 29 cases, indicating the minimally affected region and location of *DLK1-MEG3*.

The region affected by aUPD14q was variable between individuals and there was no difference between cases diagnosed with a haematological malignancy and those picked up in population-based screens (Figure 1). Since the boundary of our smallest region of aUPD14q, case G_3499, was only defined to the nearest megabase, our minimally affected region was conservatively defined by case E5364 as 11.2 Mb running from 14q32.12 to the telomere (chr14: 94,245,652-105,417,313). The minimal affected region (MAR) in previously published analyses of genome-wide association studies data was similar: 7.4 Mb (chr14: 98,962,371-qter)^4^ and 6.9 Mb (chr14: 99,425,044-qter).^3^ This region does not include *FANCM,* a variant of which was associated with aUPD14q in a single case.^18^

### Exome sequencing

Initially, with the aim of identifying a recurrently mutated 14q gene, we sequenced the whole exomes of cases with aUPD14q (*n*=7) or +14 (*n*=1). We focused on the identification of novel variants in the minimal region of aUPD14q (chr14: 94,245,652-qter), thus capturing both constitutional and somatic mutations that might provide a selective advantage when reduced to homozygosity. No gene was identified with likely causative variants in more than one individual (Supplementary Table 3). In addition, no rare variants were identified in *FANCM* which falls outside our MAR. Although it is possible that a target gene might have been missed due to inadequate coverage or mutational complexity, we considered an alternative explanation.

### Methylation bias associated with aUPD14q

**Figure 2:**
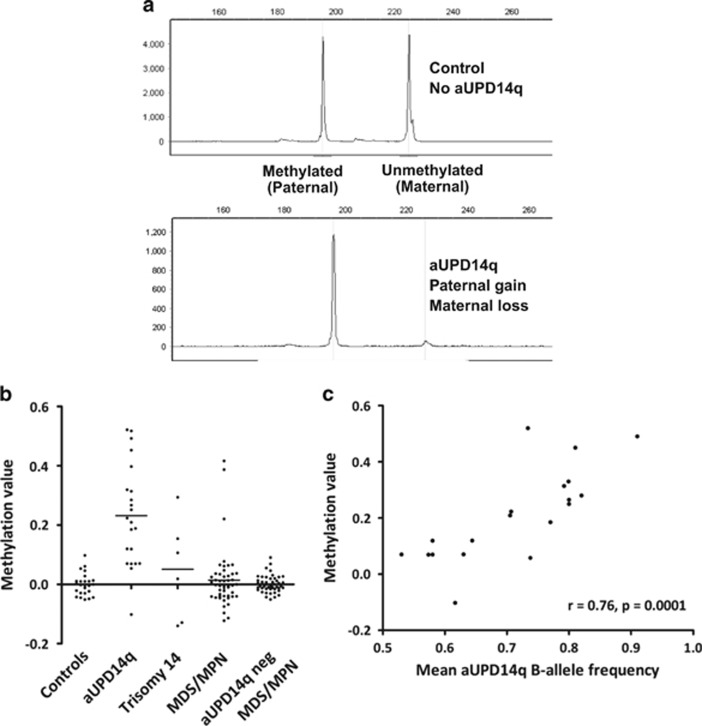
*MEG3* methylation analysis. (**a**) PCR of bisulphited DNA allowed differential amplification and relative quantification of the methylated (paternal) and unmethylated (maternal) alleles. Control samples show approximately equal paternal and maternal peak sizes, whereas samples with a high level of aUPD14 show complete loss of the maternal allele. Methylation values are calculated as the methylated paternal allele peak height divided by the sum of methylated and unmethylated peaks, with control values normalised to zero. Complete loss of the maternal allele and gain of the paternal allele in cases is seen in cases with aUPD14q in the great majority of cells and is indicated by a methylation value of 0.5. In many cases, aUPD14q is only seen in a proportion of cells, as indicated by the SNP array BAF in the affected region. Consequently, the methylation value ranges between 0 and 0.5 for paternal gain/maternal loss and between 0 and −0.5 for paternal loss/maternal gain. (**b**) Methylation values for healthy controls (*n*=24), cases with aUPD14q (*n*=22), trisomy 14 (*n*=6), randomly selected MDS/MPN cases without knowledge of their aUPD14q status (*n*=48) and MDS/MPN cases that were known to be negative for aUPD14q or other visible chromosome 14 abnormalities (*n*=48). The results show a highly significant skewing (*P*<0.0001 Mann–Whitney *U*-test) in the aUPD14q cases towards paternal aUPD. (**c**) Graph illustrating the strong correlation between the methylation value and mean aUPD BAF for each case.

Constitutional maternal UPD14q causes Temple syndrome, whereas paternal UPD14q causes Kagami–Ogata syndrome. Both are developmental conditions resulting from aberrant expression of genes in the imprinted *DLK1*-*MEG3* domain at 14q32. *DLK1*-*MEG3* is the only known imprinted locus on chromosome 14 and is retained in the aUPD14q MAR defined above. To test if *DLK1*-*MEG3* might be targeted by chromosome 14 abnormalities, we determined the *MEG3* methylation status of cases with aUPD14q (*n*=22) or +14 (*n*=6; Table 1). *MEG3* is methylated on the paternally inherited chromosome 14 but is unmethylated when maternally inherited. An increase in methylation therefore indicates paternal aUPD14q, that is, gain of the region containing *MEG3* from the paternal chromosome 14 and loss of the corresponding region from the maternal chromosome 14 (Figure 2). Samples from cases with aUPD14q showed a striking increase in methylation (mean methylation value 0.24, s.d. ±0.17) compared with healthy controls (*n*=24; mean methylated value 0.00, s.d. ±0.04) consistent with paternal aUPD (Figures 2a and b; Mann–Whitney *U*-test, *P*<0.0001). Methylation values and BAF in the region of aUPD14q were strongly correlated (Spearman's rank correlation, *r*=0.76, *P*=0.0001) (Figure 2), suggesting that the relatively low levels of methylation imbalance in some individuals was due to the presence of a small clone with aUPD14q. In contrast, methylation in the +14 cases showed no parental bias (mean methylation value 0.05, s.d. ±0.17; +14 cases versus controls, *P*=0.34, Mann–Whitney *U*-test). This is the first time to our knowledge that aUPD has been associated with a specific parent of origin effect, a finding that indicates that aUPD14q targets an imprinted locus.

### Methylation bias is specific for aUPD14q

To determine if a parent of origin effect is seen in other regions of aUPD, we analysed MDS/MPN cases with aUPD22q (*n*=5), an abnormality that is also seen recurrently in myeloid malignancies and population cohorts of elderly individuals. Like aUPD14q, the target of aUPD22q has not been identified but there is a candidate imprinted locus (*NHP2L1*) within the affected region.^19^ Methylation analysis indicated maternal aUPD22q in two cases and paternal aUPD22q in three cases (Supplementary Figure 1). Although the number of cases is small, the findings clearly indicate no parent of origin effect, supporting the notion that the imbalance observed for aUPD14q is likely to be pathogenetically relevant.

### Methylation bias in cases without aUPD14q

To determine if 14q imprinting abnormalities are more widespread in myeloid neoplasia, *MEG3* methylation status was examined in additional MDS/MPN cases (*n*=96) that had either been analysed by SNP arrays and were known to be negative for aUPD14q (*n*=48) or were randomly selected without knowledge of their aUPD14 status (*n*=48). Most cases had methylation levels that were indistinguishable from healthy controls, but three had a clear gain of methylation (Figure 2b). All three were of unknown aUPD14q status and so we tested six microsatellite loci at 14q32: only one case was homozygous at all six loci suggesting that gain of methylation in the remaining two cases had arisen by a mechanism other than aUPD14q (Supplementary Table 4).

### EZH2 mutations in some cases with aUPD14q

*MEG3* and other transcripts within the *MEG3*-*DLK1* locus have been implicated in regulation of the polycomb repressive complex 2 (PRC2),^20^ components of which are recurrently inactivated in myeloid neoplasia by mutation of *EZH2*, *SUZ12* or *EED*.^2, 21^ It is possible therefore that the consequence of aUPD14q might be functional inactivation of PRC2, in which case we would expect aUPD14q and PRC2 mutations to be mutually exclusive. Partial sequence analysis of *EZH2* and *SUZ12*, the most commonly mutated components of PRC2, revealed causative *EZH2* mutations in two out of eight aUPD14q cases (Supplementary Table 5), which suggests that functional inactivation of PRC2 is unlikely to be the primary consequence of aUPD14.

### aUPD14q may be an early or late event

Inspection of the exome sequencing data for the aUPD14q cases (*n*=7) revealed no other obvious driver mutations in three out of four individuals from the population cohorts, suggesting that aUPD14q alone may be sufficient to promote clonal haemopoiesis. The fourth individual (PIVUS 931) was positive for *JAK2* V617F as well as a *TP53* mutation; he was subsequently diagnosed with polycythemia vera following recruitment into the PIVUS study. In contrast, all three cases with diagnosed myeloid malignancy that were exome sequenced had additional somatic driver mutations in *SF3B1*, *EZH2*, *JAK2* or *TET2* (Table 1).

**Figure 3:**
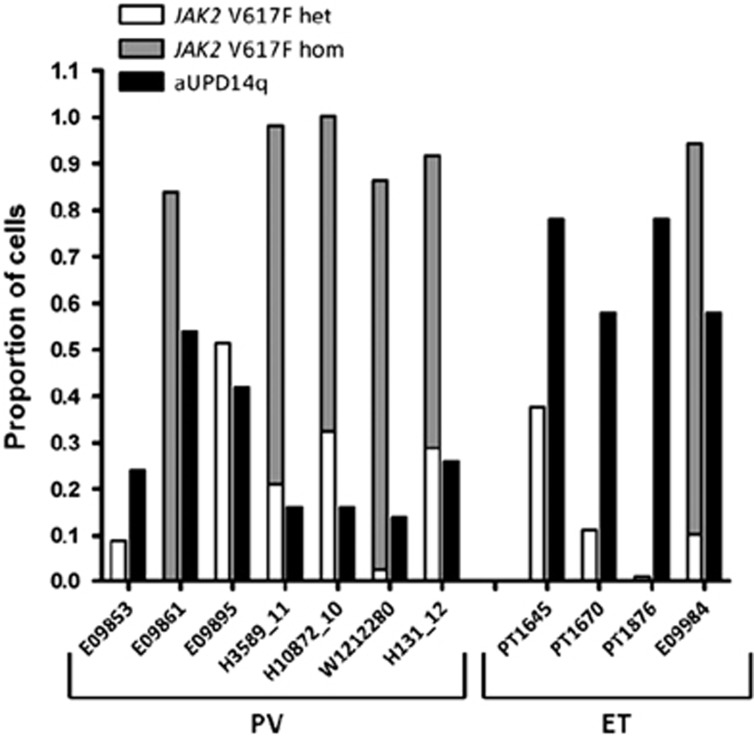
Proportions of cells positive for *JAK2* V617F and aUPD14q in cases with both abnormalities. Black bars indicate cells positive for aUPD14q; grey bars indicate homozygous *JAK2* V617F mutant cells and white bars indicate heterozygous *JAK2* V617F mutant cells.

Of the aUPD14q cases that were positive for *JAK2* V617F, information on the mutation level as well as the BAF for 9p and 14q aUPD was available for 11 individuals. For these, we were able to estimate both the proportion of cells with aUPD14q and the proportion of cells which were homozygous or heterozygous for *JAK2* V617F (Figure 3). This in turn enabled us to infer whether aUPD14q or *JAK2* V617F was likely to have arisen first. Assuming that *JAK2* V617F and aUPD14q arose within the same clone, our findings suggest that aUPD14q arose before *JAK2* V617F in cases E09853, PT1645, PT1670 and PT1876 since the proportion of cells positive for aUPD14q was twofold or higher than the proportion positive for the *JAK2* mutation. In contrast, *JAK2* V617F is likely to have arisen first in cases E09861, H3589_11, H10872_10, W1212280 H131_12 and E09984. The order is uncertain for case E09895. It is notable therefore that aUPD14q arose first in three out of four cases with essential thrombocythemia but in only one out of seven cases of polycythemia vera.

## Discussion

Developmental abnormalities arising from constitutional UPD result from inappropriate expression of imprinted genes. Inherited maternal UPD14q is associated with Temple Syndrome, characterized by low birth weight, hypotonia, motor delay, early onset of puberty and short adult stature.^22^ Inherited paternal UPD14q is associated with Kagami–Ogata syndrome, characterized by severe developmental delay, hepatoblastoma and characteristic dysmorphology.^23^ Both abnormalities result from aberrant expression of the *DLK1*-*MEG3* domain at 14q32, a large and complex imprinted cluster of genes and non-coding RNAs. The methylated paternally derived chromosome expresses the protein-coding genes *DLK1*, *RTL1* and *DIO3*, while the non-methylated maternally derived chromosome expresses the non-coding genes *MEG3*, *MEG8*, *asRTL1*, multiple miRNAs and snoRNAs.^24^ It is not known whether the observed clinical phenotypes are caused by aberrant expression of an individual gene, individual non-coding RNAs or a combination of factors.

Our finding of a highly significant parent of origin effect associated with aUPD14q strongly implicates an imprinted locus as the primary target, and that paternal homozygosity for this target provides a growth advantage over cells that harbour both alleles. Del(20q), another somatic abnormality associated with myeloid neoplasms, has been shown to target an imprinted gene cluster^25^ but this is the first time to our knowledge that aUPD has been associated with an imprinted target. Although we cannot exclude the possibility that aUPD14q might target another imprinted locus at 14q32, we believe it is highly likely that the true target is *DLK1*-*MEG3* since (i) this cluster falls with the 11.2 Mb MAR, an interval that contains 121 known protein-coding genes plus the immunoglobulin region, and (ii) despite extensive searches there are no other confirmed imprinted loci within the MAR, or indeed elsewhere on chromosome 14. Furthermore, expression of *DLK1*-*MEG3* has been reported to be deregulated in diverse neoplasms in the absence of chromosome 14 abnormalities, including acute promyelocytic leukaemia^26, 27^ and myelofibrosis.^28, 29^ In our study, we did not have suitably stored material for expression analysis of aUPD14q cases; however, expression analysis alone is unlikely to be informative since by definition aUPD would be expected to distort the normal expression of imprinted genes in the affected region whether they were of pathogenetic relevance or not.

Of the 22 aUPD14q cases analysed for *MEG3* methylation in our study, 21 had positive values indicative of paternal aUPD. One case had a negative methylation value. We do not have an explanation for this aberrant case, but potentially other mutations may have been present that promoted clonal expansion. We note that aUPD for other regions has not been associated with a specific mutational target in all cases, for example, we found that only 9 of 12 MDS/MPN cases with aUPD7q had *EZH2* mutations, with the target of the remaining 3 cases remaining unclear.^2^

Similar to other mutations associated with clonal haemopoiesis in population cohorts,^30, 31^ aUPD14q is more prevalent in the elderly. Previous studies found aUPD14q in 21/50 222 (0.04%) individuals, rising to 14/15 101 (0.09%) in those >60 years of age,^3, 4^ an increase that parallels the increase in myeloid neoplasia seen in the elderly. Analysis of longitudinal data estimated a 10-fold elevated risk of developing a haematological malignancy for individuals with any acquired chromosome anomaly,^4^ and a similar risk was estimated for individuals with somatic mutations in genes known to be associated with myeloid malignancies.^30, 31^ The most commonly mutated genes in population cohorts were *DNMT3A*, *TET2* and *ASXL1*, and the finding that most individuals had only a single abnormality suggested that mutations in these genes are often initiating events for clonal haemopoiesis. Inspection of our exome sequencing data for the aUPD14q cases revealed no mutations in known driver genes in three out of four population cohort cases. By contrast, all three cases with diagnosed myeloid malignancy had additional somatic driver mutations. We suggest therefore that aUPD14q may also initiate clonal haemopoiesis and predispose to overt malignancy. However, when we examined cases that had both aUPD14q and *JAK2* V617F, it was clear that aUPD14q may be an early event in some MPN cases but a late event in others. Furthermore, we found that aUPD14q tends to arise before *JAK2* V617F in essential thrombocythaemia but after V617F in polycythemia vera. This is reminiscent of the finding that mutated *TET2* may precede or follow the acquisition of *JAK2* V617F in MPN, with the order of acquisition influencing clinical features and stem cell biology.^32^

## Figures and Tables

**Table 1:** Summary of cases with chromosome 14 abnormalities

*Patient identifier*	*aUPD14q region or karyotype (hg19 co-ordinates)*	*aUPD14q size (Mb)*	*Disorder*	*Exome*	*Driver mutations*	*Mean B-allele frequency of aUPD14q region*	*Methylation level*^a^
E7173	chr14: 70924501-105930406	35.0	ET	Yes	*SF3B1* K700E	0.81	0.45
E5364	chr14: 94245652-105417313	11.2	CMML	Yes	*EZH2* exon9:c.729-2A>-	0.91	0.49
E6459	chr14: 33291583-105930406	72.6	PMF	Yes	*JAK2* V617F*; TET2* D1242V	0.73	0.52
PT03B08	chr14:20211644-107285437	87.1	PMF	—	*CALR* Type 2	0.82	0.28
CB44	chr14:22053729-107285437	85.2	ET	—	*CALR* Type 1	0.74	0.05
PT02B05	chr14:73672831-107274052	33.6	ET	—	*MPL* W515K	0.8	0.25
PT02E11	chr14:23582569-107220898	83.6	ET	—	*CALR* Type 2	0.8	0.27
AN804	chr14:21240673-107285437	86.0	PMF	—	*CALR* Type 1	0.80	0.32
11_4629	chr14:21209871-107287663	86.1	ET	—	*CALR* Type 2	0.71	0.21
E6430	chr14:23102969-107274052	84.2	ET	—	*CALR* Type 1	0.64	0.12
E09853	chr14:20213937-107274052	87.1	PV	—	*JAK2* V617F	0.62	−0.10
E09861	chr14:20295510-107274052	87.0	PV	—	*JAK2* V617F	0.77	0.19
E09895	chr14:56103882-107287663	51.1	PV	—	*JAK2* V617F	0.71	0.22
E09984	chr14:50192257-107287663	57.1	ET	—	*JAK2* V617F	0.79	0.31
H3589_11	chr14:59183573-107287663	48.1	PV	—	*JAK2* V617F	0.58	0.07
H10872_10	chr14:24653187-107222493	82.6	PV	—	*JAK2* V617F	0.58	0.12
W1212280	chr14:24843620-107287663	82.4	PV	—	*JAK2* V617F	0.57	0.07
H131_12	chr14:92280675-107274052	15.0	PV	—	*JAK2* V617F	0.63	0.07
PT1544	chr14: 94238353- 107287663	13.0	ET	—	*CALR* Type 2	0.69	ND
PT1645	chr14: 21070264- 105965102	84.9	ET	—	*JAK2* V617F	0.89	ND
PT1670	chr14: 23248583- 107287663	84.0	ET	—	*JAK2* V617F	0.79	ND
PT1876	chr14: 72220169- 107231967	35.0	ET	—	*JAK2* V617F	0.89	ND
G_735	chr14:27349540-107349540	80^b^	PMF	—	*JAK2* V617F	NA	0.40
G_3358	chr14:83349540-107349540	24^b^	PMF	—	*JAK2* V617F	NA	0.52
G_3499	chr14:101250540-107349540	6^b^	PMF	—	*JAK2* V617F	NA	0.19
ULSAM 546	chr14:24944467-107349540	82.4	PC	Yes	None detected	0.69	0.07
ULSAM 831	chr14:40334000-107349540	67.0	PC	Yes	None detected	0.67	ND
PIVUS 931	chr14:94156220-107331190	13.2	PC	Yes	*JAK2* V617F; *TP53* exon6:c.376-2 A>G	0.77	ND
PIVUS 892	chr14:77435975-107349540	29.9	PC	Yes	None detected	0.61	ND
E4051	+14	—	aCML	Yes			0.15
E6901	+14	—	CMML	—			−0.14
W813483	46,XX,?dup(12)(p11p12),idic(14) (p11)/47,idem,+idic(14)	—	AML	—			0.29
W1301891	47,XX,+14[20]	—	MDS	—			0.12
W1407109	46,XX,del(5)(q13q33)[1]/58,sl,+1,+2,+del(5),+8,+9,+10,+11,+13,+14,+19,+21,+22[9]/60,sdl1,+6,add(6)(q1),+mar[2]	—	MDS	—			0.11
W1409489	45,X,-Y[6]/46,idem,+14[8]/46,XY[6]	—	MDS	—			−0.13
E7820	aUPD14q by microsatellite analysis	—	CMML	—	*EZH2* C590F		

Abbreviations: aCML, atypical chronic myeloid leukemia; AML, acute myeloid leukemia; CMML, chronic myelomonocytic leukemia; ET, essential thrombocythemia; MDS, myelodysplastic syndrome; NA, not applicable; ND, not determined; PC, cases from Swedish elderly population-based cohorts with no haematological malignancy diagnosed at the time of sampling (PIVUS 931 was subsequently diagnosed with polycythemia vera); PMF, primary myelofibrosis; PV, polycythemia vera.

a Paternal chromosome loss or gain is given by the methylated (paternal) peak height divided by the sum of methylated and unmethylated peaks, normalised to shift control values to zero. A positive value indicates methylation (paternal chromosome) gain compared with controls.

b Regions of aUPD14q only defined to the nearest megabase, the minimally affected region of aUPD14q was therefore conservatively defined by case E5364 as 11.2 Mb, chr14: 94245652-qter.
