# Supplementary material for: Profound parental bias associated with chromosome 14 acquired uniparental disomy indicates targeting of an imprinted locus
Source: Leukemia. 2015 Jul 31;29(10):2069–74. doi: 10.1038/leu.2015.130 (PMC4687469; doi:10.1038/leu.2015.130)
Supplement: Supplementary Figure 1 [file leu2015130x1.ppt]

## Slide 1
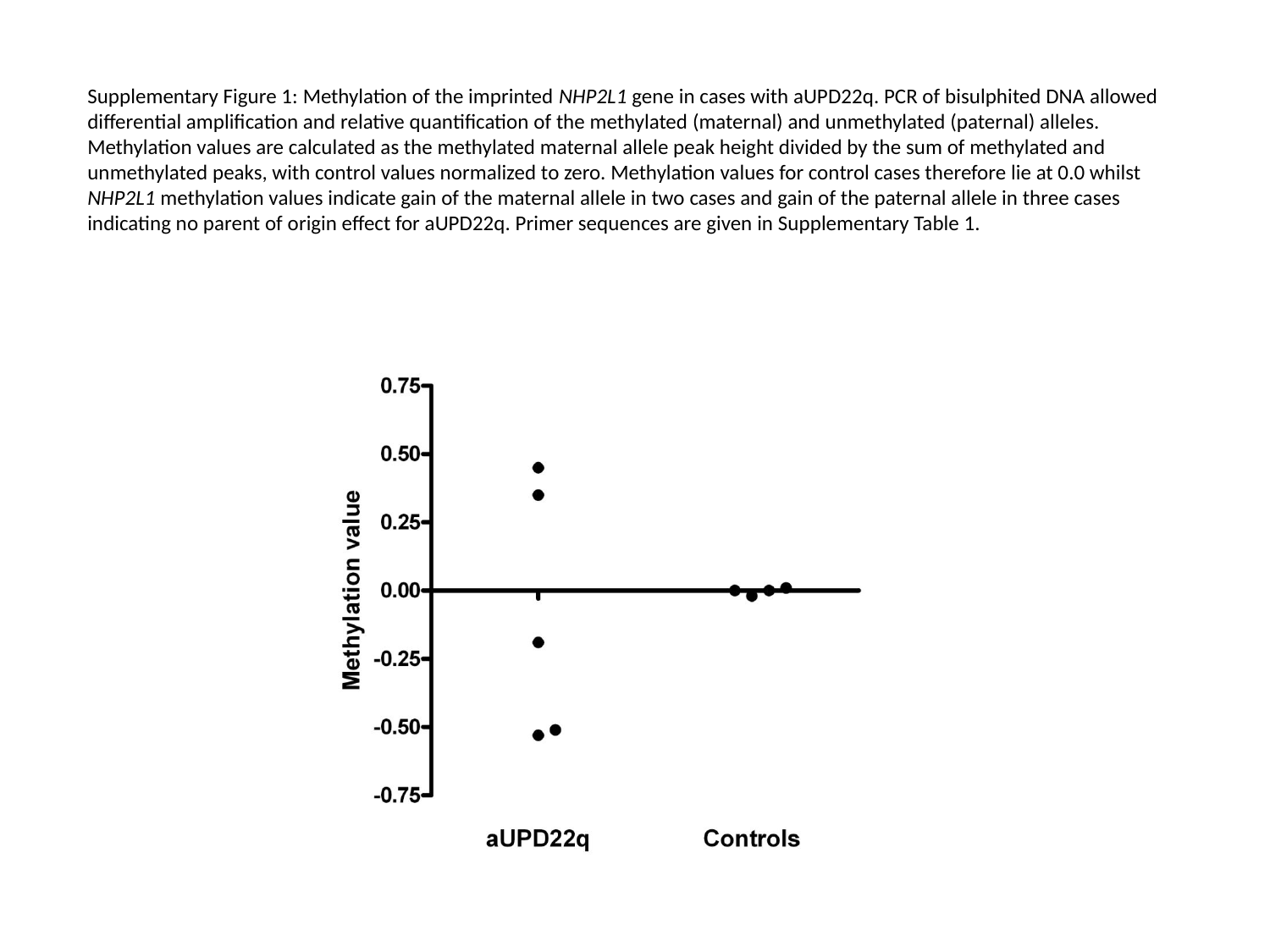

Supplementary Figure 1: Methylation of the imprinted NHP2L1 gene in cases with aUPD22q. PCR of bisulphited DNA allowed differential amplification and relative quantification of the methylated (maternal) and unmethylated (paternal) alleles. Methylation values are calculated as the methylated maternal allele peak height divided by the sum of methylated and unmethylated peaks, with control values normalized to zero. Methylation values for control cases therefore lie at 0.0 whilst NHP2L1 methylation values indicate gain of the maternal allele in two cases and gain of the paternal allele in three cases indicating no parent of origin effect for aUPD22q. Primer sequences are given in Supplementary Table 1.
