# Supplementary material for: Profound parental bias associated with chromosome 14 acquired uniparental disomy indicates targeting of an imprinted locus
Source: Leukemia. 2015 Jul 31;29(10):2069–74. doi: 10.1038/leu.2015.130 (PMC4687469; doi:10.1038/leu.2015.130)
Supplement: Supplementary Table 1 [file leu2015130x2.doc]

**Supplementary Table 1. Primers used in this study**

(i) Primer set used for *MEG3* methylation analysis

Genomic location (hg19): chr14:101292454-101292679

Methylated allele size: 226bp

Unmethylated allele size: 199bp

Methylated primer: CGCGTTTTGGTTCGTTGGTTTTGGCGGCG

Unmethylated primer: GTGTAGATGGTGGAGAGTAGAGAGGGAGTGTG

Universal FAM labelled primer: CTCCAACAACAAAACCCAAAATCAAACAAACTCTC

(ii) Primer set used for *NHP2L1* methylation analysis

Genomic location (hg19): chr22:42,078,073-42,078,275

Methylated allele size: 199

Unmethylated allele size: 209

Methylated primer: CATCGTATATAACGTACGAATCGCG

Unmethylated primer: CATATCACCATCATATATAACATACAAATCACA

Universal FAM labelled primer: GTTGTAAAAAAAYGGAAGGAGGAAAAGGTAGGTG

(iii) primers used to amplify microsatellites

| Microsatellite | Forward | Reverse | Genomic position (hg19) of PCR product |
| --- | --- | --- | --- |
| D14S553 | TACAAAGCCACAAGGGAGT | AGCTATGTTTGTGCCATGG | chr14:94336357-94336643 |
| D14S267 | TTAATGCCCACTGAATGCT | AAGGCAGCCCTGGTTT | chr14: 99224410-99224198 |
| D14S1006 | TTCCACAGGGCAAGCAGTA | TTCTGGCAAAACCCAACC | chr14:101179611-101179741 |
| D14S542 | GCCCATCAGTAGACGAACAA | GACTCCATCTCAAACCACGC | chr14:104544065-104544154 |
| D14S292 | CATGAAGGCAGCCTCA | CTGTGTGGTGCATCAATG | chr14: 104596932-104596816 |
| D14S1007 | CTCCATTCCCATACGTCC | AGCTCCTATATGTCTTCACACAG | chr14: 105978100-105977978 |
