# Supplementary material for: Profound parental bias associated with chromosome 14 acquired uniparental disomy indicates targeting of an imprinted locus
Source: Leukemia. 2015 Jul 31;29(10):2069–74. doi: 10.1038/leu.2015.130 (PMC4687469; doi:10.1038/leu.2015.130)
Supplement: Supplementary Tables [file leu2015130x4.doc]

Supplementary Table 3: Novel variants found by exome sequencing within the chromosome 14 minimal region of acquired uniparental disomy

| SAMPLE ID | CHR | LBP | RBP | Ref | Alt | Gene | Variant info | Depth | VAD | VAF | mean BAF |
| --- | --- | --- | --- | --- | --- | --- | --- | --- | --- | --- | --- |
| E6459 | 14 | 101349539 | 101349539 | G | A | *RTL1* | NM_001134888:exon1:c.C1587T:p.Y529Y | 11 | 10 | 0.909091 | 0.73 |
| PIVUS892 | 14 | 96730589 | 96730589 | C | T | *BDKRB1* | NM_000710:exon3:c.C570T:p.I190I | 270 | 103 | 0.381481 | 0.61 |
| PIVUS931 | 14 | 104196160 | 104196160 | G | A | *ZFYVE21* | NM_001198953:exon6:c.G557A:p.R186Q | 204 | 164 | 0.803922 | 0.77 |
| ULSAM831 | 14 | 104195491 | 104195491 | G | A | *ZFYVE21* | NM_024071:exon5:c.G498A:p.Q166Q | 44 | 10 | 0.227273 | 0.67 |
| ULSAM546 | 14 | 99641602 | 99641602 | G | A | *BCL11B* | NM_138576:exon4:c.C1571T:p.P524L | 66 | 32 | 0.484848 | 0.69 |

VAD=variant allele depth; VAF=Variant allele frequency; mean BAF=mean B-allele frequency within the aUPD14q MAR[EXACT REGION??].

Supplementary Table 4: Three unselected CMML samples were found to have gain of *MEG3* methylation. Microsatellite analysis was used to look for any associated aUPD14q. E7820 was homozygous at all loci tested suggesting the presence of aUPD14q; E8434 and E7795 both showed heterozygosity at loci telomeric of *DLK1-MEG3* (101-102Mb) suggesting gain of methylation had arisen by a mechanism other than by aUPD14q.

| Microsatellite marker | D14S553 | D14S267 | D14S1006 | D14S542 | D14S292 | D14S1007 | Homozygous loci count |
| --- | --- | --- | --- | --- | --- | --- | --- |
| Genomic position (hg19) | 94.3Mb | 99.2Mb | 101.2Mb | 104.5Mb | 104.6Mb | 106.0Mb |  |
| E8434 | HET | HET | HET | HOM | HET | HET | 1 |
| E7820 | HOM | HOM | HOM | HOM | HOM | HOM | 6 |
| E7795 | HET | HOM | HOM | HET | HOM | HOM | 4 |

Supplementary Table 5: Complete or selected coding exons of *EZH2* and *SUZ12* were sequenced (reference sequences: *SUZ12*, NM_015355; *EZH2*, NM_004456). No mutations in *SUZ12* were identified; two mutations in *EZH2* were found, one of which destroys an essential splice site, a second falls within the CXC domain and predicted to be damaging or possible damaging by SIFT, Mutation taster and Polyphen.

| Sample | *EZH2* exons screened | *SUZ12* exons screened | Mutations |
| --- | --- | --- | --- |
| PT03B08 | 12-19 | 15 |  |
| CB44 | 12-19 | 15 |  |
| E5364 | 2-20 | 15 | *EZH2*. c.729-2del(A) |
| E6459 | 2-20 | 15 |  |
| PT02B05 | 12-19 | 15 |  |
| PT02E11 | 12-18 | 15 |  |
| AN804 | 12-19 | 15 |  |
| E7820 | 12-19 | 15 | *EZH2*.c.1769G>T.p.590C>F |
